# Untargeted Lipidomic Reveals Potential Biomarkers in Plasma Samples for the Discrimination of Patients Affected by Parkinson’s Disease

**DOI:** 10.3390/molecules30040850

**Published:** 2025-02-12

**Authors:** Kateryna Tkachenko, Jose María González-Sáiz, Consuelo Pizarro

**Affiliations:** Department of Chemistry, University of La Rioja, 26006 Logroño, Spain; kateryna.tkachenko@unirioja.es (K.T.); josemaria.gonzalez@unirioja.es (J.M.G.-S.)

**Keywords:** untargeted metabolomics, Parkinson’s disease, patient stratification, metabolic signatures, liquid chromatography, neuro disease

## Abstract

Nowadays, the diagnosis of Parkinson’s disease (PD) remains essentially clinical, based on the subjective observations of clinicians. In addition, misdiagnosis with other neuro disorders, such as Alzheimer’s (AD), can occur. Herein, an untargeted lipidomic analysis of 75 plasma samples was performed to identify lipid species capable of discriminating between these two neuro groups. Therefore, PLS-DA and OPLS-DA analysis revealed significant differences in patient profiles in the sphingolipid and glycerophospholipid categories. As a result, a putative lipid biomarker panel was developed, which included HexCer (40:1; O2) and PC (O-32:0), with an area under the receiver operating characteristic curve (AUC) > 80, respectively. This panel was effective in discriminating between diseased and healthy subjects, but most importantly, it could discriminate between two neurodegenerative disorders that can present similar symptoms, namely PD and AD. Together, these findings suggest that the dysregulated metabolism of lipids plays a critical role in AD and PD pathology and may represent a valuable clinical tool for their diagnosis. Thus, further targeted studies are encouraged to better understand the underlying mechanisms of PD and confirm the diagnostic potency of the identified lipid metabolites.

## 1. Introduction

Nowadays, the prevalence of progressive neurodegenerative disease (ND) constitutes a socio-economic burden due to the ageing population and improved longevity [1]. NDs are characterized by the progressive loss of the specific nerve cells in the central nervous system (CNS). Thus, Parkinson’s disease (PD) is the second most complex progressive neurodegenerative disorder after Alzheimer’s disease (AD), with a broad range of motor and non-motor symptoms [2]. PD is characterized by the accumulation of intracellular protein aggregates, Lewy bodies, composed primarily of the protein alpha-synuclein [3]. For this reason, PD is both a cerebral amyloid disease and the most common synucleinopathy [4]. In recent years, the scientific community’s interest in finding specific biomarkers of these neurodegenerative diseases has grown substantially [5]. Despite insight derived from causative genetic mutations, which explain only a small proportion of cases, the remaining 90% are due to non-genetic factors and are sporadic [6]. The exact pathogenetic mechanism underlying these diseases is still poorly understood.

Despite all the advances in genetics and neuroimaging, most ND diagnosis remains essentially clinical, based on the subjective observations of clinicians. The most critical challenge in clinical practice is the inability to make a definitive diagnosis at the early stages and predict disease initiation or progression. Usually, the signs and symptoms of neurodegeneration appear later in the disease when the neurodegenerative process has started and is irreversible. In addition, even when the new diagnostic criteria are correctly applied, the misdiagnosis rate is still high [7], precluding intervention at the early stage of the disease. Cognitive impairment is the main symptom of Alzheimer’s disease and also the primary non-motor symptom of Parkinson’s disease [8,9]. Thus, it is extremely important to perform comprehensive clinical evaluations, including detailed patient history, neurological examinations, and advanced imaging techniques, to differentiate between PD and AD accurately [10]. In addition, accurate diagnosis is essential for implementing appropriate treatment strategies and improving patient outcomes.

Moreover, the clinical frame could be further complicated by the increasing incidence of comorbidities. Therefore, the molecular mechanisms leading to neurodegeneration remain elusive.

Among current hypotheses, a complex convergence of genetic and environmental factors, such as exposure to heavy metals, smoking, or dietary habits, has been proposed to play an important role in PD pathogenesis [11]. Many studies have highlighted the role of oxidative stress and its elevated implication in protein misfolding, death of neuronal cells, and lipid peroxidation—all mechanisms at the basis of neuron degeneration [12]. Increasing evidence has demonstrated that brain and cognitive ageing are accompanied by peripheral metabolic perturbations [13]. Since metabolites are the end points of multiple interactions and processes that happen in the organism, metabolomics is an increasingly recognized tool for investigating the altered metabolic profiles of patients. Moreover, within the metabolome, lipids are involved in important biological functions, including the structure of cell membranes, energy storage, and signaling. Thus, many studies have reported that glycerophospholipids, sphingolipids, and ceramides exert important biological roles in the central nervous system (CNS), such as signal transduction, apoptosis, and structural neuronal maintenance [14]. In addition, changes in total ceramide molecular species and even changes in ceramide acyl chain length can affect membrane order or membrane lipid peroxidation [15]. Recently, it was shown that phospholipids and sphingolipids are highly concentrated in membrane lipids rafts (MLRs). These specialized plasma membrane microdomains are integral to regulating intracellular trafficking and signal transduction [16]. Therefore, alterations in the composition of MLRs have been reported in PD. Likewise, it was shown that Alzheimer’s patients are characterized by decreased levels of sphingomyelins and increased levels of ceramides due to sphingomyelin hydrolysis [17]. Together, these findings suggest that dysregulated metabolism of the lipids plays a critical role in AD and PD pathology and may represent a valuable clinical tool for their diagnosis.

Metabolomics studies have already proven their great potential coupled with high-throughput techniques to perform metabolic profiling and evaluate significantly discriminative biomarkers between healthy and diseased groups that may contribute to neurodegeneration [18,19,20,21,22,23,24]. Moreover, the high-throughput capabilities of modern MS systems enable the analysis of large sample cohorts, enhancing the statistical power of studies and the reliability of the potential biomarkers identified [25]. When compared to traditional clinical diagnostic methods, untargeted lipidomics offers a more detailed molecular insight into disease mechanisms. While conventional diagnostics often rely on symptomatic assessments and limited biochemical tests, lipidomics can uncover specific lipid alterations that precede clinical manifestations, potentially leading to earlier detection and intervention [26]. In addition, since blood is a readily available and easy to collect sample compared to cerebrospinal fluid (CSF), which could create complications during the collection step, many studies opt to investigate blood biomarkers.

Thus, herein, we performed an untargeted lipidomic analysis using the UPLC-MS/MS technique on plasma samples to investigate the possible biomarkers responsible for changes and disarrangement in the pathogenesis of NDs such as AD and PD. Many studies focus only on comparing the metabolic profiles between diseased and healthy controls. In this study, patients with PD and AD were studied, since misdiagnosis between Parkinson-related dementia and Alzheimer’s dementia still occurs. Lipid extraction was performed to select only the lipid species possibly involved in PD or AD pathogenetic mechanisms.

Traditionally, diagnoses are based on clinicians’ subjective observations and scoring systems, often conducted during initial patient visits. While effective, this approach can be complemented by integrating lipidomics. By analyzing lipid profiles, clinicians can gain deeper insights into disease mechanisms, potentially leading to earlier and more accurate diagnosis. Incorporating specific panels of identified molecular compounds can enhance clinical interventions and improve patient outcomes [27].

## 2. Results

### 2.1. Differentiation Between PD, AD, and Healthy Controls

Firstly, to exclude the presence of possible patterns or correlations between sex, age, and patient subgroups, a metadata heatmap was performed. Any specific correlation or trend was observed in our patient cohort (Figure S1). Therefore, global lipidome profiling of CO samples and PD- and AD-diseased patients was conducted to investigate how lipids are affected in diseased patients.

**Figure 1:**
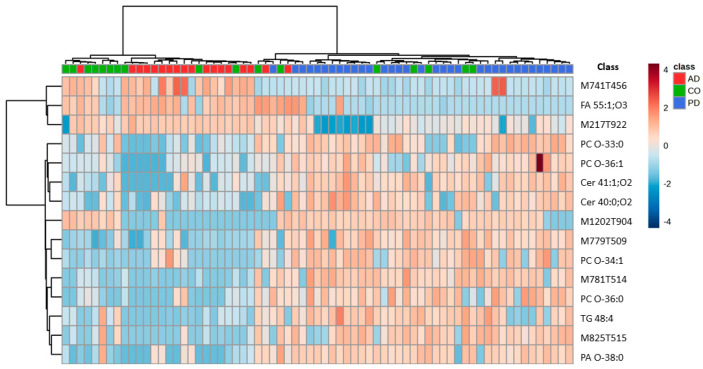
Heatmap plot displaying patient clustering (control samples (CO), Parkinson’s, and Alzheimer’s patients (PD and AD), respectively) according to one-way ANOVA followed by Tukey’s post-test (*p* < 0.05; FDR-adjusted). Lipid species identified are arranged in rows, while samples are organized in columns based on cluster analysis, utilizing Euclidean distance and the Ward clustering algorithm. In the heatmap plot, each colored cell represents values above (red) or below (blue) the mean normalized peak intensity for a specific compound. Abbreviations: Cer: ceramides; FA: fatty acids; PA: phosphatidic acids; PC: phosphatidylcholines; TG: triglycerides.

A one-way ANOVA univariate analysis identified 88 lipid molecular species that showed significant alterations across all groups. The 15 most discriminative features with tentative identification of lipids are displayed in the heatmap plot (Figure 1). Of note, a tiny amount of clustering is observed between the groups; most controls are dispersed between the two diseased groups. This result could be explained by the presence of possible alteration of the lipidomic profile among controls similar to those patients affected by PD or AD.

**Figure 2:**
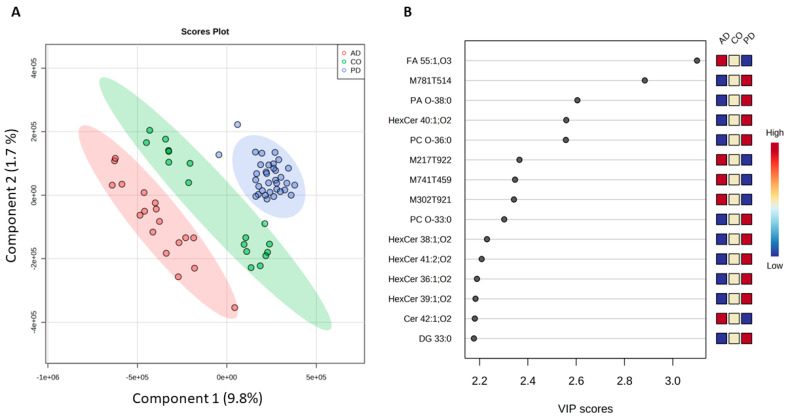
Multivariate analysis of plasma lipidome from healthy controls (CO), Parkinson’s (PD), and Alzheimer’s (AD) diseased patients. (**A**) Score plot of partial least square–discriminant analysis (PLS-DA); (**B**) Top 20 identified lipid compounds according to component 1 values of the PLS-DA model. Abbreviations: Cer: ceramides; DG: diacylglycerols; FA: fatty acids; PA: phosphatidic acids; PC: phosphatidylcholines.

In addition, PLS-DA was performed (R2 = 0.95 and relatively high Q2 = 0.49), and the score plot of the PLS-DA showed a relatively clear group clustering according to component 1 (9.8%) (Figure 2). As revealed by the top 15 identified features selected by the VIP score, the lipid trend observed while performing ANOVA is repeated.

Thus, the alterations in several molecular species seem to belong to sphingolipids and glycerophospholipids, triglycerides, and fatty acids metabolism, and the alteration in such lipid species is evidenced by both univariate and multivariate analyses.

Unfortunately, some features that contribute to group differentiation remained unidentified. But those that we tentatively identified are perfectly in line with previous data, revealing the differences between the PD and AD lipid profiles. Therefore, among the 15 most discriminative features based on the VIP score, FA 55:1, O3 (VIP > 3) seems to contribute majorly to patients’ differentiation. Compounds such as PA O-38:0 and PC O-36:0 and various ceramide derivates were also selected as the most discriminative.

Therefore, we conducted a pairwise comparison to investigate the lipid differences among specific groups.

### 2.2. Pairwise Comparison

**Figure 3:**
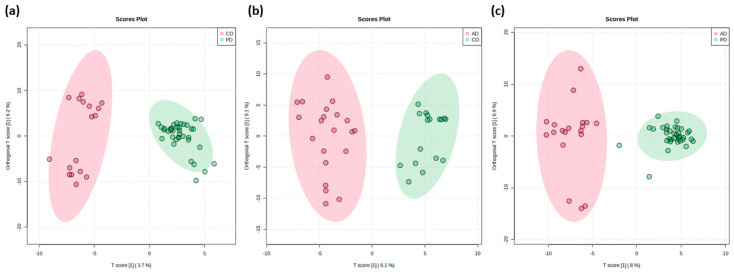
Pairwise comparison by orthogonal partial least square–discriminant analysis (OPLS-DA) score plot of the UPLS-MS/MS data in ESI (-) mode. Before statistical analysis, the data were Pareto-scaled. Score plot of the OPLS-DA revealing a clear segregation of groups: (**a**) healthy controls and Parkinson’s patients, (**b**) controls and Alzheimer patients, and (**c**) Alzheimer’s and Parkinson’s patients.

To explore the metabolic differences between groups, three binary classifications were performed using the supervised OPLS-DA method, which maximizes the distance between groups and identifies essential variables to the classification based on the VIP score. A cross-validation algorithm validated each separation, showing relatively high R2 and Q2 values, and an increased performance from components 1 to 3 was observed. As shown in Figure 3, all the groups are spatially segregated from one another. Thus, the control group was separated from the diseased groups: CO vs. PD (R2Y = 0.69 and Q2 = 0.32) and CO vs. AD (R2Y = 0.62 and Q2 = 0.32), respectively. In addition, PD and AD patients were also significantly separated (R2Y = 0.74 and Q2 = 0.59).

According to the score OPLS-DA plots, the control and Alzheimer’s groups were less homogeneous than the Parkinson’s group. This heterogeneity could be explained by the possible presence of other comorbidities in this group of patients.

**Figure 4:**
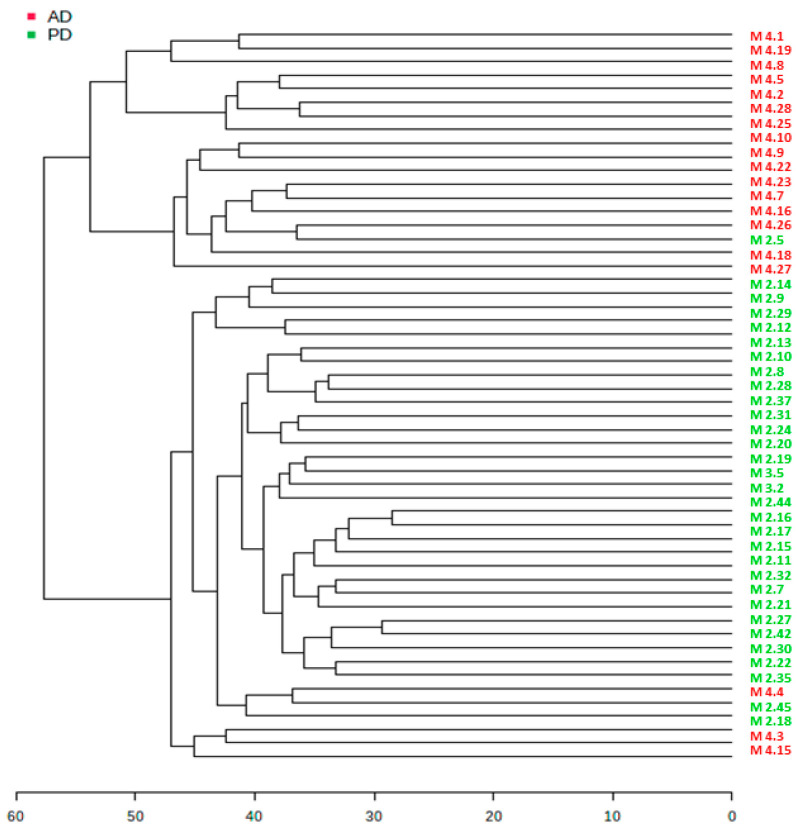
Hierarchical clustering dendrogram of AD and PD samples using the Euclidian distance measure and the Ward clustering algorithm.

In addition, a clear separation between PD and AD samples can be observed in the dendrogram obtained by performing the hierarchical clustering (HC) algorithm (Figure 4), with a Euclidean distance measure (in the scale distance from 1 to 60) and the Ward clustering algorithm. As observed, some overlapping samples are present, which could indicate the similarities in the metabolic profile between Parkinson’s patients and Alzheimer’s patients.

Differences in lipidome alterations evidenced by OPLS-DA models must be interpreted cautiously, since univariate statistics yielded fewer lipid features (Figure 3). Therefore, an attempted assignment was also performed for these compounds based on the minimum m/z error and the most probable neutral loss.

The obtained features reinforce the importance of the PLS-DA results when comparing three groups, confirming the involvement of sphingolipid and glycerophospholipid metabolism in PD and AD diseases. A panel of the most significant putatively identified compounds are summarized in Table 1.

**Figure 5:**
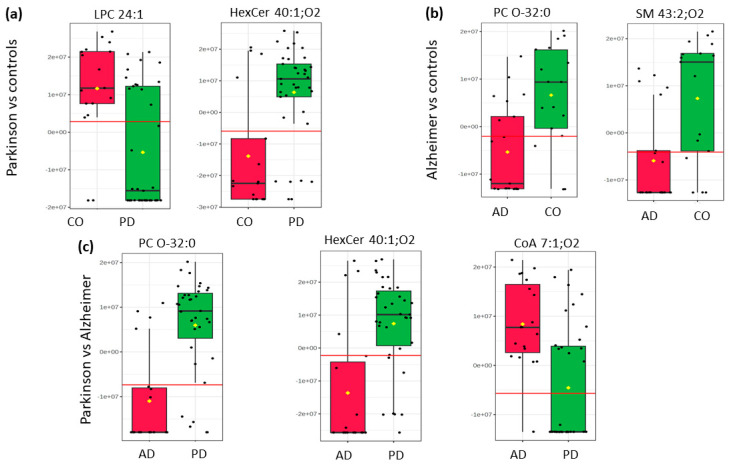
Box plots highlighting relative abundances of key lipid species that distinguish (**a**) Parkinson’s disease (PD) from healthy controls (CO), (**b**) Alzheimer’s disease (AD) from controls, and (**c**) Parkinson’s from Alzheimer’s. Each subpanel illustrates two or three representative lipid markers selected for their discriminative power. Black dots represent individual sample data points, whereas yellow diamonds mark group means. Red reference lines across each panel represent a classification threshold.

Thus, ceramide HexCer (40:1; O2) was discriminative in both pairwise comparisons, namely to differentiate Parkinson’s patients and controls and Parkinson’s and Alzheimer’s subjects. Of note, most samples belonging to the Parkinson’s group displayed increased levels of HexCer (40:1; O2) when compared to CO (log2(FC) = −1.3118) or AD (log2(FC) = −1.4582), suggesting that ceramide metabolism may play a key role in PD pathology. Likewise, the phosphatidylcholine PC (O-32:0) showed the most noticeable change (*p*-value < 0.05) and decreased levels in AD patients when compared to PD (log2(FC) = −1.7798) or controls (log2(FC) = −1.3693). Interestingly, the PD group showed increased levels of PC (O-32:0) compared to the AD group and reduced levels of LPC 24:1 when compared to controls. On the other hand, the putative biomarker sphingomyelin SM (43:2; O2), a ceramide biochemical precursor, was under-expressed in the AD group compared to controls, suggesting a possible association between sphingolipid metabolism and Alzheimer’s disease. Finally, the statistical significance and high VIP score (2.24) suggest that the putative compound CoA 7:1; O4 is an important biomarker for differentiating PD from AD. This fatty acyl is significantly higher in AD compared to PD (positive log2(FC) = 1.258), suggesting an underlying metabolic mechanism that involves the higher expression of this compound in AD but not in PD pathogenesis. Nevertheless, these results suggest that a panel combining multiple lipid biomarkers may enhance diagnostic accuracy compared to using a single lipid species. The levels of biomarker expression between groups can be visualized in the box plot (Figure 5).

**Figure 6:**
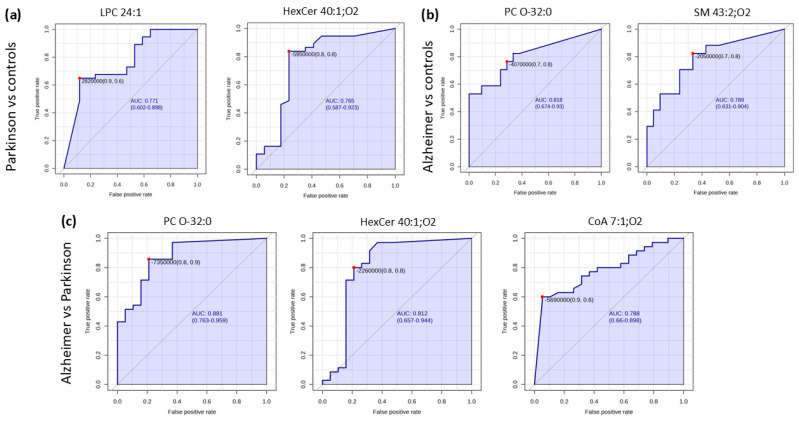
The diagnostic performance of identified lipids via AUC curves are indicated for comparison between (**a**) healthy controls and Parkinson’s patients, (**b**) controls and Alzheimer’s patients, and (**c**) Alzheimer’s and Parkinson’s patients. The AUC, 95% CI of each biomarker’s sensitivity (true positive rate), and specificity (false positive rate) are displayed. The red dot on each curve indicates the optimal threshold (cut-off) that provides the best balance between sensitivity and specificity.

The AUC values of the ROC curves of the potential lipid biomarkers were calculated to validate the discriminating power of the compounds responsible for separating controls from diseased patients and distinguishing groups with neurodegenerative disorders. Higher values of AUC close to 1 indicate higher prediction. Significant AUC values (above 70) were observed in all biomarkers by performing binary classifications (Figure 6). Thus, the diagnostic performance of potential biomarkers is summarized in Table 2.

As noted, all the AUC models showed a relatively high diagnostic performance. By comparing the PD vs. CO groups, LPC 24:1 (AUC = 0.771, 95% CI: 0.602–0.898) and HexCer 40:1;O2 (AUC = 0.765, 95% CI: 0.587–0.923) both showed a moderate discrimination ability. Likewise, SM 43:2;O2 (AUC = 0.789, 95% CI: 0.631–0.904) seems to have moderate diagnostic potential between the AD and CO groups. Nevertheless, PC O-32:0 (AUC = 0.818, 95 CI: 0.674–0.93) showed a strong ability to separate AD from CO. In addition, PC O-32:0 takes part of the biomarker panel to discriminate between Parkinson’s and Alzheimer’s disorders, reaching the highest AUC values (AUC = 0.874, 95% CI: 0.763–0.959) among other comparative models, also confirming its importance in AD pathogenesis. In addition, the biomarker CoA 7:1; O4 from the panel PD vs. AD showed the highest specificity values (94.70%) among all other discriminative lipid metabolites, suggesting its important contribution to PD and AD segregation. Moreover, the putative metabolite HexCer 40:1;O2, already selected to discriminate PD vs. CO, showed a good diagnostic performance in classifying PD and AD groups (AUC = 0.765, 95% CI: 0.587–0.923).

These results confirm that the identified compounds, mainly choline-dependent phospholipids and ceramides, hold great promise as biomarkers to diagnose neurodegenerative diseases.

## 3. Materials and Methods

### 3.1. Chemicals

All aqueous solutions were prepared using ultrapure water obtained from the Milli-Q system (Millipore, Bedford, MA, USA). LC-MS grade acetonitrile (ACN), isopropyl alcohol, ammonium formate, high-performance liquid chromatography (HPLC)-grade methanol, and methyl-ter-butyl ether (MTBE) were sourced from Aldrich Chemie (Steinheim, Germany).

### 3.2. Study Population

Blood from overnight fasting subjects was collected at the Molecular Neurobiology Department at the Biomedical Research Center of La Rioja (CIBIR), and the plasma of each participant was obtained. The study was conducted on 45 patients with PD, 27 patients with AD, and 25 normal elderly control (CO) subjects, who belong to the family environment and are of a similar age to the patients with a diagnosed disease (Table 3). The study was performed in compliance with the principles outlined in the Helsinki Declaration and received approval from the Ethics Committee of San Pedro Hospital. Written informed consent was obtained from all participants. Any additional information was included in this study.

To assess the distribution of our data across the study categories an initial Power Analysis was performed, confirming that the data followed a normal distribution, as detailed in the Supplementary Materials (Figure S2).

### 3.3. Collection and Handling of Plasma Samples

Blood samples were collected in Becton Dickinson (BD) Vacutainer plastic tubes (Madrid, Spain) with K_2_EDTA for plasma separation. Thus, the plasma fraction was centrifuged at 2200× *g* for 15 min at 4 °C. All samples as aliquots of 200 μL were stored in Eppendorf tubes at −80 °C until further use.

### 3.4. Lipid Extraction

Before lipid extraction, plasma thawing was performed according to the ultrasound-assisted extraction (USAE) protocol for lipidomic analysis [28]. Thus, the samples left to defrost in the fridge for 8 h overnight were submitted to extraction the next day.

Following the MTBE-US-assisted lipid extraction method, a 10 μL aliquot of human blood plasma was mixed with 5 μL of Milli-Q water. To precipitate proteins, 20 μL of methanol was then added, followed by vortex-mixing for 2 min. Subsequently, 250 μL of MTBE was introduced and dispersed by placing the mixture in an ultrasonic water bath from ATU Ultrasonidos (Valencia, Spain). The ultrasound frequency and power were set at 40 kHz and 100 W, respectively. The process was carried out at a temperature of 15 °C for 30 min. After ultrasonication, 25 μL of Milli-Q water was added to the mixture. Finally, the organic phase was separated by centrifugation at 3000 rpm for 10 min at 10 °C in an Eppendorf 5403 Refrigerated Centrifuge (Hettich, Tuttlingen, Germany).

The lipid extracts in the upper phase were diluted five times with injection solvent before being collected and poured into an autosampler vial. Quality samples (QC) were processed identically to the study samples.

### 3.5. Liquid Chromatography-Mass Spectrometry Analysis

To determine plasma lipid profiles, a Waters Acquity UPLC chromatography system (Milford, MA, USA) was used, equipped with a Waters Acquity HSS T3 100 × 2.1 (i.d.) mm 1.8 μm particle size column and a Waters VanGuard precolumn of the same material. To ensure the quality and stability of samples, the temperature of the autosampler was maintained at 5 °C and the column at 55 °C. Elution was carried out using a gradient mobile phase composed of phase A (a 60:40 *v*/*v* acetonitrile–water mixture containing 10 mM ammonium formate) and phase B (a 10:90 *v*/*v* acetonitrile–isopropanol mixture with 10 mM ammonium formate). Chromatographic separation was achieved through a linear gradient, increasing from 40% to 100% B over 10 min, followed by a 2 min hold at 100% B. Subsequently, phase A was raised from 0% to 60% within 3.5 min. The total analysis time was 15.5 min, with a flow rate of 0.4 mL/min.

Mass spectrometry data were collected using a Waters Synapt XS HDMS (Waters Corp, Milford, USA) in continuum mode with electrospray ionization (ESI+/−) across a mass range of m/z 50–2000. The capillary voltage was set to 1.75 kV, while the sampling cone voltage was adjusted to 40 V. The source and desolvation temperatures were maintained at 120 °C and 500 °C, respectively. Gas flow rates were configured at 800 L/h for desolvation gas and 50 L/h for cone gas, with the nebulizer gas pressure set to 6 bars. Data acquisition was performed in resolution mode with a scan time of 0.4 s, and fragment ion details were obtained using a collision energy ramp ranging from 20 to 50 V.

Lockmass correction was achieved by infusing leucine enkephalin at 10 µL/min through a lockspray probe and acquired every 30 s; for positive mode, [M + H] ^+^ = 556.2771, and for negative mode, [M − H] ^−^ = 554.2615. The data were collected using MassLynx V 4.2 (Waters Corp., Milford, MA, USA).

The samples and controls were alternated concerning the run, avoiding the batch effect. Moreover, QC samples were inserted regularly throughout the analytical run (after every 10 actual samples) to check the methodology’s performance and test its precision.

### 3.6. Data Processing

The raw mass data were imported to Progenesis QI software (version 2.4, nonlinear dynamics) (Waters Corporation, Milford, MA, USA) for data processing. Thus, peak picking was performed based on peak detection, alignment, retention time correction, and normalization. Dimensionality reduction was imperative, considering the bigger number of variables than the sample size. An unsupervised principal component analysis (PCA) was performed on generated pre-processed data to monitor the stability of the study and observe the samples’ separation and exclude the presence of outliers. Thus, QC samples as references were used to monitor the analytical performance. The high degree of aggregation of the QC samples in the PCA model represents an instrumental stability and reproducibility index.

Lipid metabolites were manually characterized by their precise masses, distinctive fragments, and/or specific neutral losses [26]. The statistically significant metabolites were searched in the LIPID MAPS database, HMDB database, and METLIN database. Thus, an attempted assignment of possible features to specific compounds was performed.

### 3.7. Statistical Analysis

The generated matrices in Progenesis were subsequently analyzed using MetaboAnalyst 5.0 [29], a comprehensive free and publicly accessible metabolomics analysis platform. One-way parametric ANOVA followed by Tukey’s post-test (*p* < 0.05) was used to identify significantly altered lipid species between groups. The Benjamini–Hochberg-based false discovery rate (FDR) was used for multiple testing corrections, with *p* < 0.05 considered statistically significant. In addition, a fold change (log2FC) analysis was also performed by comparing the mean intensity of the peaks.

Thus, the traditional supervised PLS-DA method was performed to compare three groups of patients (PD vs. AD vs. CO) and the OPLS-DA analysis was applied on normalized and Pareto-scaled matrix data to perform binary classifications: (i) CO vs. PD, (ii) PD vs. AD, and (iii) CO vs. AD. Discriminative lipid features were defined based on variable importance projection (VIP) > 1. Each model was validated by Q2Y (predictive variation) and R2Y (explained variation) parameters-based leave one out cross-validation (LOOCV).

The receiver operating curves (ROCs) were obtained to validate the discriminating power of the compounds responsible for each classification. ROC curve analysis is widely considered to be the most objective and statistically valid method for biomarker performance evaluation. Thus, the area under the curve (AUC) values (>70, *p*-value < 0.05) allowed the evaluation of the sensitivity and specificity of each identified compound to be considered as a relevant biomarker.

## 4. Discussion

Lipids play a crucial role in the CNS, contributing to membrane fluidity, synaptic stabilization, and the transmission of electrical signals [30,31,32]. Consequently, dysregulated lipid metabolism has been linked to various neurodegenerative diseases, including Parkinson’s and Alzheimer’s diseases and many other injuries of the CNS [14].

We evaluated and compared the lipidomic profile of patients affected by neurodegenerative diseases such as Parkinson’s and Alzheimer’s. To the best of our knowledge, few studies have focused on both neuro disorders. Usually, very little importance is assigned to their distinction because PD and AD present overlapping symptoms and could share similar metabolic profiles, but this can lead to misdiagnosis [8,9,10]. Most previous studies focused only on the differences between one neurodegenerative disorder and control samples and, in lucky cases, on the progression of the same disease. Herein, the power of advanced untargeted metabolomic analyses based on LC-MS/MS was applied to gain insight into PD and AD differential lipid biomarkers. Blood-based biomarkers are still not routinely implemented in clinical practice but may be helpful since there is less risk of complication in older patients than with CSF sampling. By performing binary classification, the metabolites with high missingness or known drug metabolites were excluded as non-informative. Therefore, only a few statistically relevant lipid metabolites were identified and considered prognostic. In addition, we performed a tentative assignment of the compounds. Our findings highlight alterations in the sphingolipid and glycerophospholipid categories in both diseases. Among these, two compounds—HexCer (40:1; O2) and PC (O-32:0)—emerged consistently as discriminative in different classification steps, reinforcing their significance for PD and AD differentiation.

For instance, lower PC levels in AD patients were observed by performing both binary classifications: AD vs. CO and AD vs. PD, respectively. Decreased plasma PC levels in AD patients have been described previously. The targeted study by Mapstone et al. [33] showed that the pre-clinical group of AD patients would have a depletion of PC metabolites in the future. Likewise, Kim et al. [34] identified a panel of three PCs that were decreased in the plasma of (younger and older) AD participants compared to normal controls. This evidence, joined with our findings, reinforces the theory that peripheral lipids are implicated in AD pathology.

On the other hand, PC’s spontaneous hydrolysis or enzymatic degradation is known to be responsible for lysophosphatidylcholine (LPC) generation [35]. It was shown that PC and LPC mechanism alterations are implicated in many diseases [36,37,38], including both disorders under study [39]. Recently, the study of Miletić Vukajlović et al. [40] investigated the association of the PC/LPC ratio and the stages or disease progression of Parkinson’s disease, confirming that PD patients had elevated PC/LPC ratios regardless of the stage or duration of the disease. It was assumed that oxidative stress might induce modifications in the enzymes responsible for the conversion of the investigated species. In addition, López de Frutos et al. [41] found elevated serum PC levels with simultaneously decreased LPC levels, resulting in an increased PC/LPC ratio. These findings are perfectly congruent with the obtained results in our study. For instance, PC levels were increased in the PD group when comparing PD and AD patients. As previously demonstrated by other researchers [23], this difference may be explained by the fact that patients with Alzheimer’s disease are more likely to experience PC depletion as the disease progresses. Meanwhile, the LPC levels were decreased, comparing PD to normal controls. Globally, it could confirm the theory of an elevated plasma PC/LPC ratio in Parkinson’s disorder.

Similar to the controversial presence of phosphocholines in PD and AD patients, we also found different levels of ceramide derivatives between neurodegenerative disorders. Multiple lines of evidence suggest that different sphingolipids play a role in neuronal signaling and toxicity [42,43]. For instance, SM participates in signal transduction and the regulation of inflammatory processes, including the response to oxidative stress [44]. The hydrolysis of sphingomyelin generates ceramides, which are recognized for mediating the connection between Aβ pathology and neurodegeneration. Therefore, the perturbation in SM/Cer homeostasis might contribute to neurodegeneration in AD [45]. The lipidomic plasma analysis of Baloni et al. [46] identified the SM ratio as a strong intermediate trait for sphingolipid dysregulation in AD. Herein, the AD group has shown a reduced sphingomyelin level compared to healthy controls, suggesting that our findings are perfectly consistent with the literature evidence [18] and confirming the relationship between sphingomyelins and AD cognitive impairment.

Commonly, elevated Cer levels are observed in aging brains and are linked to impaired receptor trafficking and synapse loss. In addition, Cer accumulation can induce neuronal degeneration through apoptosis, autophagy, and inflammation [47]. Usually, decreased Cer levels contribute to AD disorders; meanwhile, increased levels of ceramide species (e.g., mohesylceramides or lactosylceramides) contribute to PD pathophysiology [48]. For example, mutations in the GBA gene, which encodes an enzyme crucial for Cer metabolism, are associated with increased PD risk, highlighting the role of Cer in PD pathogenesis [49]. The GBA gene, encoding for the lysosomal enzyme glucocerebrosidase, catalyzes ceramide synthesis from glucosylceramides (GlcCer), leading to the accumulation of GlcCer in the brain and blood [50]. Thus, Cer/ GlcCer ratio alterations could contribute to alpha-synuclein accumulation in glial cells [51]. In our study, the identified ceramide HexCer (40:1; O2) possibly showed higher levels in PD than controls or AD patients. Cer accumulation may impair mitochondrial function, leading to increased oxidative stress and neuronal damage; thus, it is expected that the same levels of ceramides will be found in both neuro disorders. Nevertheless, it was shown that an increase in ceramide levels is detected during the initial stage of dementia and decreases afterwards during the progression of AD [52]. Understanding these lipid alterations provides insight into the shared metabolic disturbances in PD and AD, offering potential avenues for therapeutic intervention.

Interestingly, among three compounds identified to discriminate AD from PD patients, the CoA 7:1; O4 biomarker was evaluated. Acetyl-CoA plays a key role in the proper functioning of the cell, such as glycolysis, fatty acid synthesis, or the tricarboxylic acid (TCA) cycle in mitochondria [53]. Thus, dysregulation and deficit in mitochondrial metabolism are often linked to cognitive dysfunction in AD patients. Meanwhile, increased acetyl-CoA levels provide neuroprotection [54].

Different studies have produced inconsistent findings regarding the clinical and demographic characteristics of patient subgroups based on lipid levels. Some research suggests a potential association between gender and lipid profiles; for instance, glycerophospholipids have been linked to the female sex in Alzheimer’s disease (AD) patients. Conversely, recent studies indicate that AD patients and control groups often exhibit similar lipid profiles when considering factors such as age, sex, medication use, and comorbidities [23]. Additionally, no significant differences in the phosphatidylcholine/lysophosphatidylcholine (PC/LPC) ratio [40] have been observed between genders or across different age groups. In the context of Parkinson’s disease (PD), the metabolism of dopamine analogs and agonists may vary between sexes [55]. To mitigate these effects, studies with large sample sizes of drug-naïve patients are necessary. Further, research with substantial cohorts is essential to validate these findings and elucidate the detailed pathogenesis of lipid disturbances in PD development across different sexes.

Summarizing, the identification of specific panel of lipid compounds has the potential to shape diagnostic protocols across neurodegenerative diseases, impacting healthcare practices on a larger scale. Our findings suggest that targeting Cer metabolism could provide therapeutic opportunities for both PD and AD. Modulating enzymes such as GBA or Cer synthases might mitigate lipid-driven neurodegeneration. The PC/LPC ratio holds promise as a biomarker for early diagnosis and disease progression monitoring in neurodegenerative conditions. By incorporating a larger cohort, this research addresses the need for less invasive, more accessible testing methods, which could reduce healthcare costs and patient burden. The identification of a metabolic profile for PD offers the potential for faster, more accurate diagnosis, improving patient outcomes and quality of life.

Limits of the study

Although only some specific PD and AD biomarkers were identified using an untargeted lipidomic approach, the current findings are perfectly congruent with previously reported studies. Therefore, our findings could be the standpoint for hypothesis-driven targeted analysis. Nevertheless, certain limitations should be taken into consideration. While our finding supports the validity of our statistical tests, we recognize that the reduced sample size may still constrain the study’s ability to detect subtle yet meaningful differences in lipid profiles. Thus, we recommend cautious interpretation of these results.

A larger sample size and a more homogenous cohort of participants are required to confirm our results and reduce the risk of false-positive findings.

Furthermore, the information about the effects of genetic background, nutrition, and other factors that might impact blood lipid composition could better elucidate the obtained results. Thus, for example, comorbid conditions such as diabetes and cardiovascular disease can significantly influence plasma lipid profiles, particularly in older populations, and can confound lipidomic analyses in studies of neurodegenerative diseases like Parkinson’s and Alzheimer’s diseases [56]. For instance, individuals with diabetes frequently present with dyslipidemia, which is marked by high triglycerides, elevated low-density lipoprotein (LDL) cholesterol, and reduced high-density lipoprotein (HDL) cholesterol levels. This lipid imbalance contributes to a higher risk of atherosclerotic cardiovascular disease [57]. We are not aware of the presence of comorbidities in our patient cohort, but, it is crucial to account for these comorbid conditions when interpreting lipidomic data to ensure that observed lipid changes are accurately attributed to neurodegenerative processes rather than underlying metabolic disorders. Recognizing and adjusting for these influences is essential in further studies investigating the lipidomic underpinnings of neurodegenerative diseases to avoid confounding results.

In addition, many evaluated features remain unidentified, indicating that the endogenous metabolites are still poorly understood and/or that the databases do not encompass all possible lipid species, especially novel or less common ones. This limitation can hinder the identification of certain lipids present in a sample. We are also aware about limitations of the annotation software; therefore, we highlighted that the annotation is putative.

## 5. Conclusions

In conclusion, our study demonstrated that alterations in plasma lipid metabolites could be critical in PD and AD pathogenesis. The lipidomic analysis presented herein has provided a putative biomarker panel for patient differentiation. Thus, significant changes in sphingolipids and glycerophospholipids were observed to differentiate healthy controls from diseased patients and, most importantly, to separate PD from AD. The identified lipid species could be useful to perform rapid clinical screening and avoid misdiagnosis. Further targeted studies with larger sample data sets are encouraged to confirm the robustness of our findings.

## Figures and Tables

**Table 1:** The information on selected biomarker panels. Top putatively identified lipid metabolites via MS/MS, exhibiting the greatest degree of change in plasma samples when comparing (a) PD vs. AD; (b) AD vs. CO, and (c) PD vs. CO.

*m*/*z*	±/*m*/*z*	Name	Ion	Category	*t* Stat	*p* Value	log2(FC)	VIP Score
**PD vs. AD**								
782.6632	0.0117	HexCer 40:1;O2	[M − H]^−^	Sphingolipids	−4.57	0.00003	−1.4582	2.43
764.5892	0.0081	PC O-32:0	[M + Formate]^−^	Glycerophospholipids	−5.46	0.00000	−1.7798	2.21
312.7256	0.0103	CoA 7:1;O4	[M − 3H]^3−^	Fatty Acyls	4.20	0.00011	1.258	2.24
**CO vs. AD**								
811.6900	0.0202	SM 43:2;O2	[M − CH_3_]^−^	Phosphosphingolipids	−3.65	0.00083	−1.3194	2.00
764.5892	0.0081	PC O-32:0	[M + Formate]^−^	Glycerophosphocholines	−3.54	0.00114	−1.3693	1.91
**CO vs. PD**								
782.6633	0.0117	HexCer 40:1;O2	[M − H]^−^	Sphingolipids	−4.25	0.00009	−1.3118	2.63
301.7632	0.0494	LPC 24:1	[M − 2H]^2−^	Ceramides	3.85	0.00032	1.1784	2.54

**Table 2:** Results of diagnostic performance of the putative metabolite panel in pairwise comparison: Alzheimer’s and Parkinson’s patients; controls and Alzheimer patients; healthy controls and Parkinson’s subjects.

Compound	AUC	Sensitivity	Specificity
**PD vs. AD**			
HexCer 40:1;O2	0.806	80.00%	78.00%
PC O-32:0	0.874	77.10%	78.90%
CoA 7:1;O4	0.782	60.00%	94.70%
**CO vs. AD**			
SM 43:2;O2	0.787	70.00%	76.20%
PC O-32:0	0.808	70.00%	76.20%
**CO vs. PD**			
Hex Cer 40:1;O2	0.765	78.40%	76.50%
LPC 24:1	0.768	62.20%	88.20%

**Table 3:** Distribution of patient population.

	Gender	N·	Age Distribution	Mean Age
Controls	Male	9	60–80	69 ± 1
	Female	16	43–76	59 ± 1
PD	Male	29	46–82	67 ± 1
	Female	16	59–85	67 ± 1
AD	Male	6	68–81	76 ± 1
	Female	21	60–85	73 ± 1

## Data Availability

The original contributions presented in this study are included in the article, and further inquiries can be directed to the corresponding author/s.

## Supplementary Materials

The following supporting information can be downloaded at: https://www.mdpi.com/article/10.3390/molecules30040850/s1, Figure S1: Hierarchical clustering heatmap of sample-level metadata showing how age, sex, and patient category vary across individual subjects; Figure S2: Power (and normality) assessment of test statistics from pairwise comparisons among three conditions: (A) Alzheimer’s disease (AD) vs. controls (CO), (B) AD vs. Parkinson’s disease (PD), and (C) PD vs. CO.
