# Supplementary material for: Untargeted Lipidomic Reveals Potential Biomarkers in Plasma Samples for the Discrimination of Patients Affected by Parkinson’s Disease
Source: Molecules. 2025 Feb 12;30(4):850. doi: 10.3390/molecules30040850 (PMC11857942; doi:10.3390/molecules30040850)
Supplement: Supplementary file 1 [file molecules-30-00850-s001.zip › molecules-3412688-supplementary.pdf]

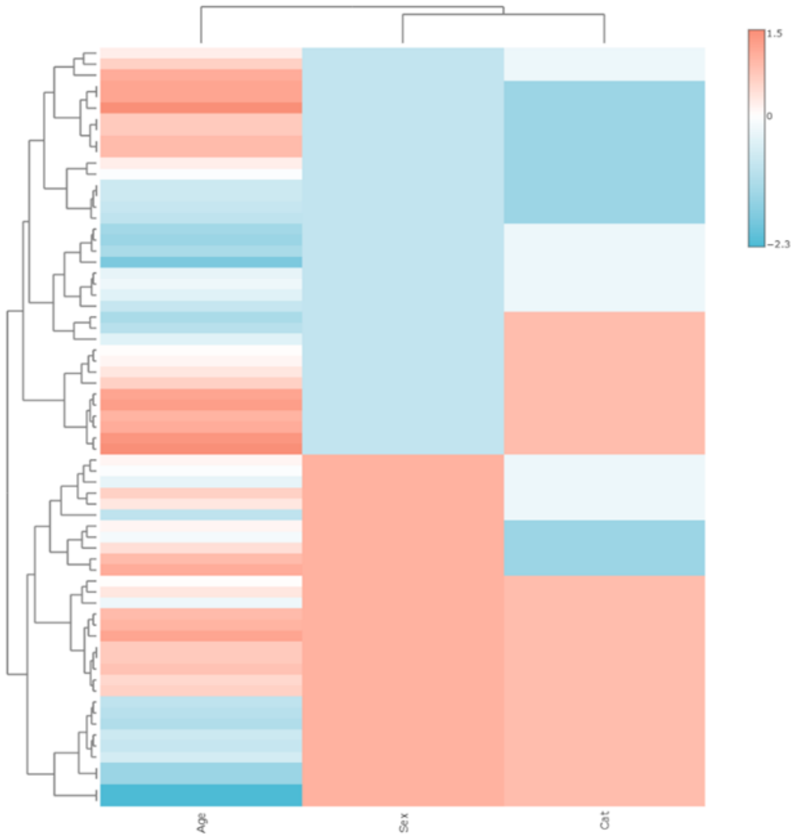

Figure S1. Hierarchical clustering heatmap of sample-level metadata showing how age, sex, and patient category vary across individual subjects. Each row corresponds to one study participant, while each column represents a specific metadata variable. The color scale ranges from approximately −2.3 (blue) to +1.5 (red), reflecting standardized values or relative intensities (e.g., older vs. younger age, female vs. male sex, and different patient categories). The dendrogram at left groups participants by similarity of metadata profiles, allowing visual identification of patterns such as clustering by age group or disease category. This heatmap facilitates an at-a-glance comparison of metadata distribution and potential correlations among key demographic and clinical variables.

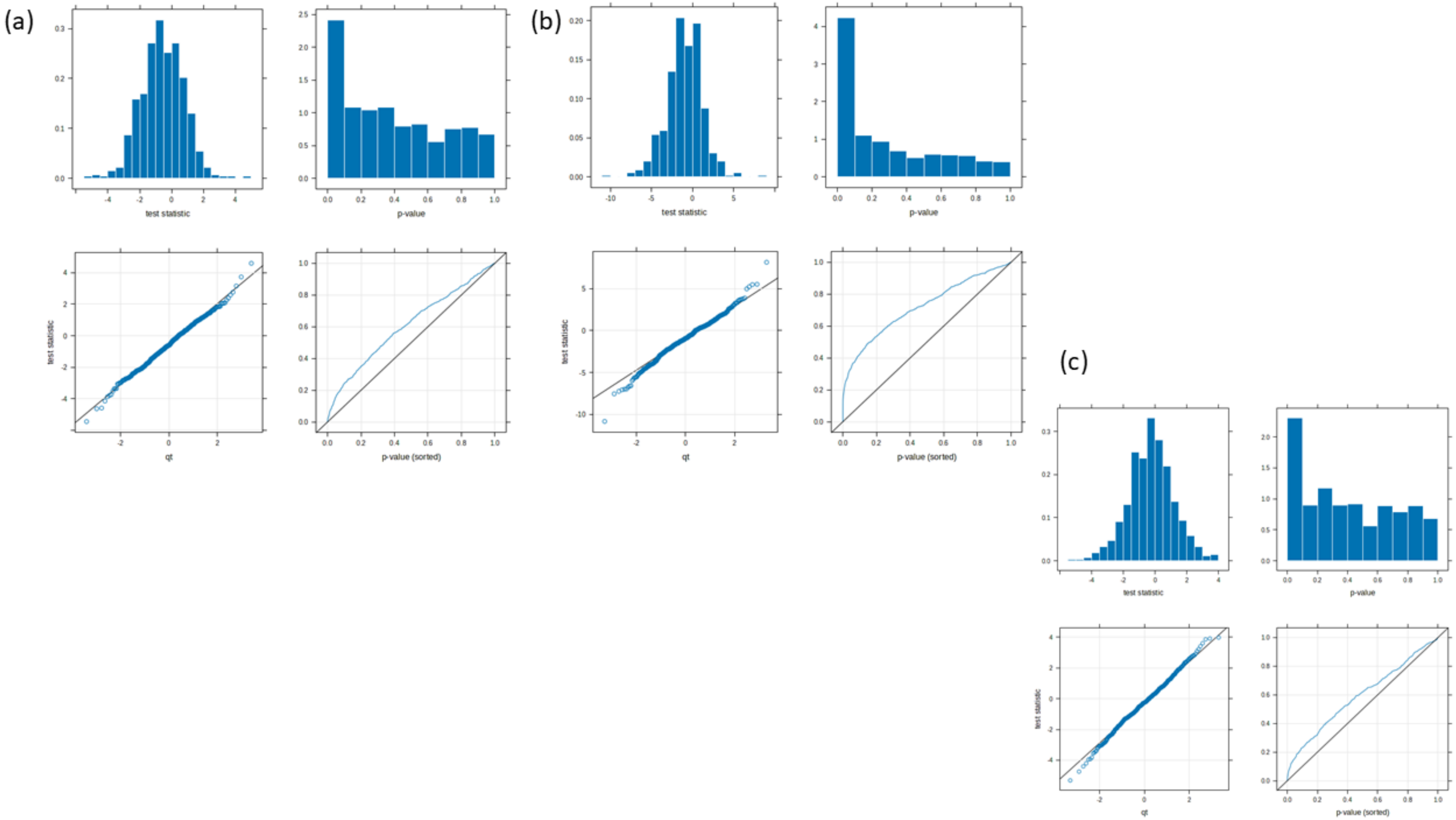

Figure S2. Power (and normality) assessment of test statistics from pairwise comparisons among three conditions: (A) Alzheimer's disease (AD) vs. controls (CO), (B) AD vs. Parkinson's disease (PD), and (C) PD vs. CO. In each subfigure, the top-left histogram shows the distribution of the test statistic (e.g., t-values), where a roughly bell-shaped curve suggests that the data follow a normal pattern. The top-right histogram displays the distribution of the resulting p-values. The bottom-left Q-Q plot compares the empirical quantiles of the test statistics against the theoretical quantiles of a normal distribution—alignment with the diagonal line indicates that the test statistics conform to normality. Finally, the bottom-right Q-Q plot visualizes sorted p-values against their theoretical expectation; proximity of points to the diagonal implies no major deviations from the uniform distribution expected under the null hypothesis. Taken together, these plots help evaluate whether test assumptions are satisfied and whether the statistical power is sufficient for the given comparisons.
